# Science during crisis and the Arnold Berliner Award 2020

**DOI:** 10.1007/s00114-020-01689-8

**Published:** 2020-08-12

**Authors:** Matthias Waltert

**Affiliations:** Conservation Biology/Workgroup on Endangered Species, Faculty of Biology and Psychology, University of Göttingen, Bürgerstrasse 50, 37073 Göttingen, Germany

The global COVID-19 pandemic has led to an unprecedented global health crisis with a terrible human toll. It has also caused enormous disruption to our society, and the scientific community has been no exception. With the closure of universities and research institutes and the implementation of lockdown measures to slow down the virus spread, many researchers have had to put their research projects on hold. Many of us in the scientific community have experienced considerable challenges to adapt to the new situation. In universities, professors have faced challenges in the sudden and forced adaption of their study programs and teaching to virtual formats. Others have struggled to adapt to home-office work, with those having children to look after and home-school experiencing the biggest challenges. Data shows that this has had a more significant impact on female researchers, whose publishing success has dropped after the closure of schools (https://www.timeshighereducation.com/news/pandemic-lockdown-holding-back-female-academics-data-show). We are well aware that many of you, as authors, reviewers, and editors have also been affected by these disruptions. Thus, we want to thank all of you for helping us to continue covering and sharing important scientific findings.

The greater negative impact on productivity among female researchers is just one of the effects of the pandemic, which also exposed many other existing social problems. Protests against other social injustices have gained particular and much-needed momentum. After the killing of George Floyd, protesters took streets and social media to demand change against historical injustices. These protests have already led to symbolic achievements such as the removal of statues of slave traders and colonialists or the release of statements by companies, institutions, and governments backing up the demands of protesters. But the movement may be having a less evident but more profound impact too, by forcing all of us to reflect on racial biases and prejudices and the existing institutional racism. As the editorial team, we also want to take this opportunity to condemn all forms of racism and say that “Black Lives Matter.”

Despite all the recent negative developments and current despair, we believe that there is room for hope. The current situation is providing some critical lessons that could help address our many other current and future crises: Above all, we can learn from it that science may overcome a crisis and even prevent it. Scientists have been warning for decades on the risks of pandemics like the current one (e.g., Cheng et al. 2007). But their claims remained in the scientific spheres and were broadly ignored by policy-makers. We are finally seeing some of our governments turning to the scientific community to seek answers and following their advice even when this requires making hard decisions. Who would have imagined, before 2019, that drastic measures such as mandatory social distancing, restricted freedom of assembly, or restricted movement could have been introduced by our governments and broadly adopted by all of us—mainly based on scientific advice? Some months after the outbreak of the global COVID-19 pandemic, we are starting to see that most of the countries which seriously and promptly embraced the recommendations of scientists are faring relatively better than those that failed to do so. However, it is still unclear whether the lessons learned from this crisis, including the need to listen to science and invest in crisis prevention, will also be applied to other pressing global crises, in particular some of the most considerable challenges of our generation: climate change and the biodiversity crisis.

In this regard, previous research has shown that deadly zoonotic diseases such as Ebola or SARS-CoV-1 are a consequence of how we, humans, are interacting with our ecosystems (e.g., Rulli et al. 2017). Deforestation, habitat degradation, climate change, and wildlife trade are known to be associated with current and future zoonotic and vector-borne diseases (e.g., Guerra et al. 2006, Chomel et al. 2007, Gottwalt 2013, Myers et al. 2013, Guégan et al. 2020). Nevertheless, these drivers of disease spread still are to receive political attention adequately. It is also highly likely that new zoonotic diseases with potentially similar or even worse effects will appear in the future unless we are able to stop the improper handling and trade of wildlife species such as bats, monkeys, and civets. We need to build green economies to protect our climate but also argue for increased spending for protecting the last rainforests on our globe, not only for the sake of preventing diseases but also to avoid future crisis caused by the loss and degradation of the functioning of the ecosystems we all depend upon. It is my sincere wish that we find enough publicity to turn facts into policies.

**Fig. 1:**
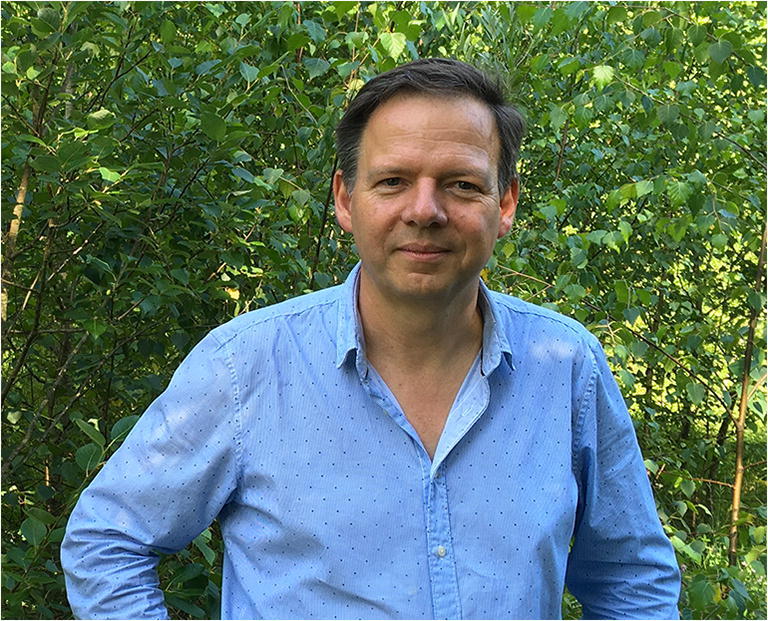
Dr. Frank Glaw, the winner of the 2020 Arnold Berliner Award

**Fig. 2:**
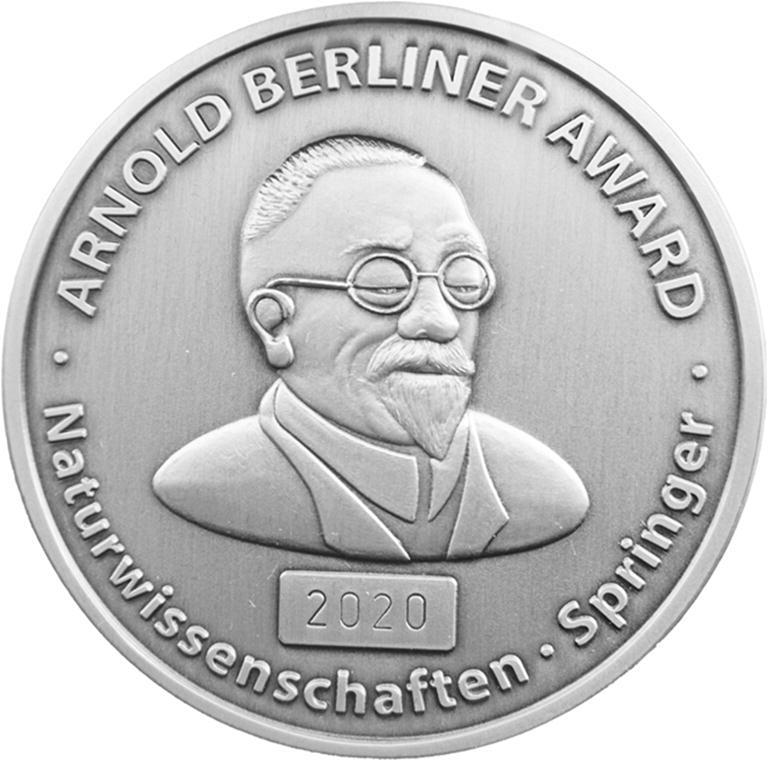
The 2020 Arnold Berliner Award medal. Arnold Berliner (1862–1942) was the founding editor of The Science of Nature (formerly Naturwissenschaften) and the journal’s editor in chief from 1913 to 1935

I also want to take this opportunity to announce the winner of the 2020 Arnold Berliner Award. *The Science of Nature* grants the Arnold Berliner Award to the lead authors of articles distinguished by their excellent, original, and—especially—interdisciplinary research. As such, the awarded articles ideally reflect the vision of Arnold Berliner (Autrum 1988; Thatje 2018). The award is sponsored by Springer and includes the Arnold Berliner Award medal (Fig. 1), a biennial subscription to the journal’s electronic edition, a 500-Euro voucher for Springer ebooks, and a cash prize of 250 Euro. I am very proud to announce that, this year, the board of editors has decided to award Frank Glaw (Fig. 2) for his article “Integrative evidence confirms new endemic island frogs and transmarine dispersal of amphibians between Madagascar and Mayotte (Comoros archipelago).” Together with co-authors Oliver Hawlitschek, Kathrin Glaw, and Miguel Vences, Frank Glaw described two new species of frogs for the Comoro Islands and shed further light on the phenomenon of overseas dispersal by amphibians (Glaw et al. 2019). Dispersal of amphibians to oceanic islands is a rare phenomenon due to the negative effect of salinity on their eggs, which already had been thought about by Charles Darwin. The study shows that there were at least two different events of overseas dispersal in the studied amphibians and also describes their ecological predispositions facilitating this dispersal. The authors apply an integrative and interdisciplinary approach by combining morphological, bioacoustic, and genetic evidence for their taxonomic work, therefore representing well one key criterion of the award. The study has also been covered by many websites, and our editors believe that it has excellent potential to become a textbook case. On behalf of the board of editors, I congratulate Frank Glaw on the award.
